# Sphingosine-1 Phosphate: A New Modulator of Immune Plasticity in the Tumor Microenvironment

**DOI:** 10.3389/fonc.2016.00218

**Published:** 2016-10-17

**Authors:** Yamila I. Rodriguez, Ludmila E. Campos, Melina G. Castro, Ahmed Aladhami, Carole A. Oskeritzian, Sergio E. Alvarez

**Affiliations:** ^1^Instituto Multidisciplinario de Investigaciones Biológicas San Luis (IMIBIO-SL) CONICET, San Luis, Argentina; ^2^Department of Pathology, Microbiology and Immunology, University of South Carolina School of Medicine, Columbia, SC, USA; ^3^Universidad Nacional de San Luis, San Luis, Argentina

**Keywords:** sphingosine-1-phosphate, tumor microenvironment, inflammation, metastasis, macrophage polarization

## Abstract

In the last 15 years, increasing evidences demonstrate a strong link between sphingosine-1-phosphate (S1P) and both normal physiology and progression of different diseases, including cancer and inflammation. Indeed, numerous studies show that tissue levels of this sphingolipid metabolite are augmented in many cancers, affecting survival, proliferation, angiogenesis, and metastatic spread. Recent insights into the possible role of S1P as a therapeutic target has attracted enormous attention and opened new opportunities in this evolving field. In this review, we will focus on the role of S1P in cancer, with particular emphasis in new developments that highlight the many functions of this sphingolipid in the tumor microenvironment. We will discuss how S1P modulates phenotypic plasticity of macrophages and mast cells, tumor-induced immune evasion, differentiation and survival of immune cells in the tumor milieu, interaction between cancer and stromal cells, and hypoxic response.

## Sphingosine-1-Phosphate

**Figure 1:**
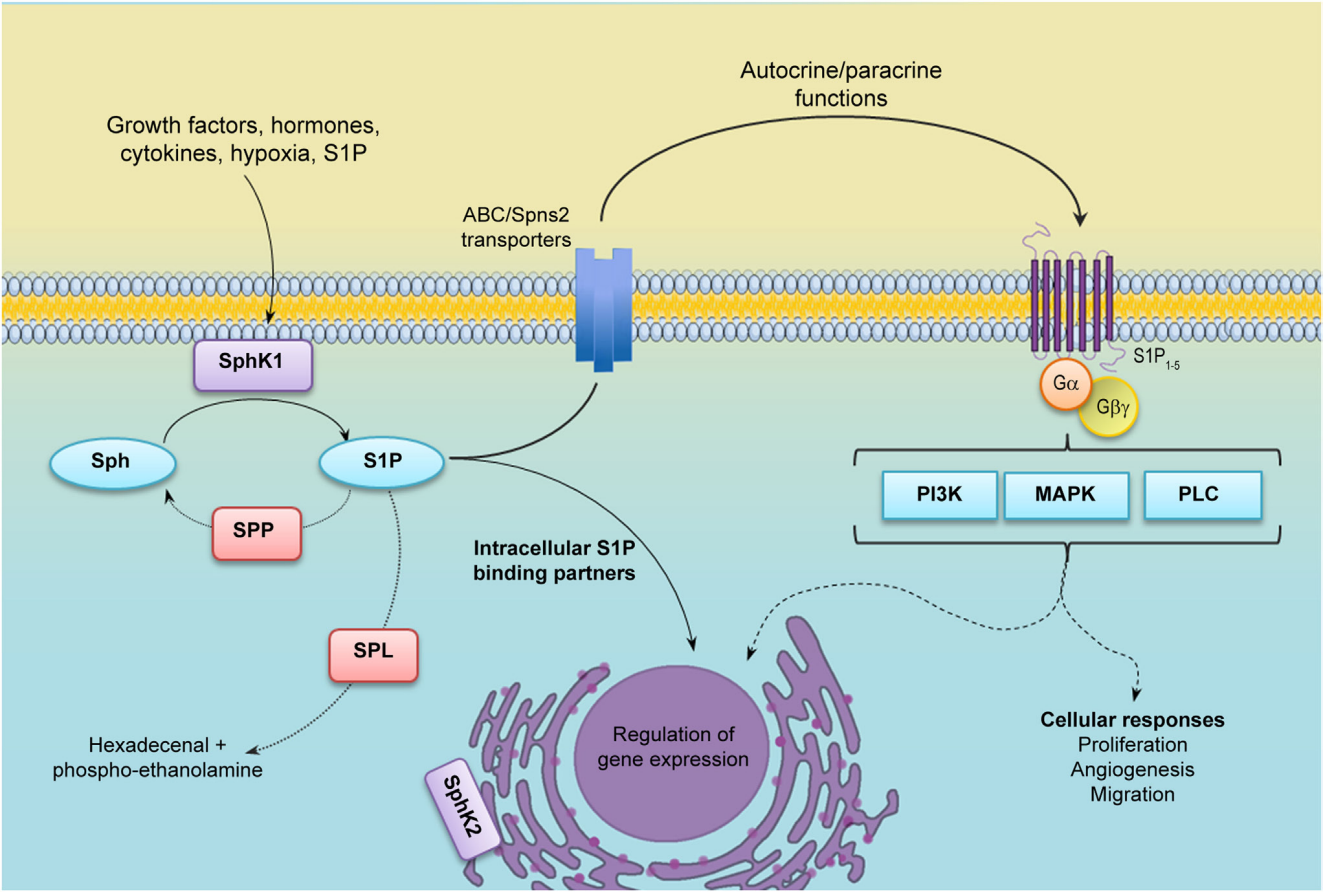
**S1P metabolism and inside-out signaling**. Many agonists stimulate cytosolic sphingosine kinase 1 (SphK1), which translocates to the plasma membrane and uses sphingosine as a substrate to generate Sphingosine-1-phosphate (S1P). S1P may be irreversibly degraded by S1P lyase (SPL) or dephosphorylated by S1P phosphatases (SPPs). After being secreted out by specific transporters, S1P binds and activates S1P receptors (S1PR1–5) in an autocrine or paracrine manner and regulates many cellular functions (inside-out signaling). S1P may also function as an intracellular second messenger through the binding of different intracellular partners. Abbreviations: PI3K, phosphatidylinositol 3-kinase; MAPK, mitogen-activated protein kinases; PLC, phospholipase C.

Sphingosine-1-phosphate (S1P) is a bioactive sphingolipid metabolite that regulates several physiological processes, including cell growth, survival, migration, differentiation, activation, and immune responses. Considering the diversity of the actions of S1P, it is predictable that deregulation of its many functions may result in the development of pathological conditions. Certainly, S1P plays important roles in cancer and disorders of the immune system. Intracellular S1P levels are tightly regulated by the equilibrium between its formation, catalyzed by sphingosine kinases (SphKs), and degradation, catalyzed by S1P lyase (SPL) and S1P phosphatases (SPPs). Two isozymes of mammalian SphK have been cloned and characterized, SphK types 1 and 2 (SphK1 and SphK2) (1). Interestingly, S1P can be exported out of the cell either by the specific transporter Spinster 2 (Spns2) (2) or by several members of the ABC transporter family (3). In turn, S1P exerts extracellular functions through the binding to five specific G-protein-coupled receptors (GPCR), named S1P receptors (S1PR) 1–5 (1, 4). This autocrine and/or paracrine action of S1P is known as “inside-out signaling” (1, 5) and is critical for a great variety of cellular responses. Although most of the known actions of S1P are mediated by S1PRs, in the last few years, it has become evident that S1P also exerts intracellular functions by targeting different molecules (Figure 1).

## The Complexity of S1P Signaling

Many distinct agonists, including cytokines, growth factors, and hormones, among others, induce translocation of SphK1 to the plasma membrane, where its substrate sphingosine resides, leading to the activation of the enzyme. SphK1 translocation depends on its phosphorylation at Ser 225 by ERK1/2 (6) and interaction with calcium and integrin-binding protein 1 (CIB1) (7). Indeed, a mutation of SphK1 on Ser225 residue behaves as a dominant negative, although the protein maintains its kinase activity (6). SphK1 translocation also results in the production of S1P in a location that allows its extracellular export, making it readily available for ligation of S1PRs to trigger the “inside-out” signaling. On the other hand, SphK2 is located in several organelles, including the nucleus and endoplasmic reticulum (Figure 1). Although much less is known about SphK2 activation (8), it is also phosphorylated and activated by ERK1 (9). Likewise, SphK2 is phosphorylated by protein kinase D (PKD) in HeLa cells, leading to nuclear export and cytoplasm accumulation of the enzyme (10).

S1P receptors are coupled to distinct heterotrimeric G proteins leading to downstream activation of diverse effector pathways, including phospholipase C (PLC), phosphatidylinositol 3-kinase (PI3K), and mitogen-activated protein kinases (MAPK), among others (11). The functional response of each cell to S1P varies depending on its S1P receptor repertoire that is also modulated by several signals (12).

S1P receptor-1 has a central role in cell migration and it is undoubtedly involved in angiogenesis and immune cell trafficking. A gradient of S1P characterizes physiological conditions, where S1P levels are elevated in blood and lymph and low in tissues. This gradient is important for vascular integrity and allows immune cell egress from lymphoid organs to the circulation (13). Furthermore, high S1P levels produced during inflammatory processes drive immune cell recruitment to inflamed tissues. During embryonic development (14) and tumor progression, the S1P/S1PR1 axis promotes endothelial precursor recruitment and vascular development. The crucial role of S1PR1 in lymphocyte migration culminated by the rapid development of FTY-720 (fingolimod) as an immunosuppressive drug, approved by the Food and Drug Administration (FDA) for the treatment of recurrent form of multiple sclerosis (MS). FTY-720 induces S1PR1 endocytosis and degradation, thus preventing lymphocyte egress and inflammation, a hallmark of MS (15).

For the most part, binding of S1PR1 has been shown to promote migration of several cancer cell lines (16–19), while S1PR2 exerted opposite effect (20–22). Indeed, stimulation (16) or inhibition (23) of S1P-induced migration in melanoma cells depends on expression of S1PR1 or S1PR2, respectively. However, some reports also established a positive role of S1PR2 in migration (24, 25), indicative of a complex role of S1PRs in cell motility. Similar to S1PR1, engagement of S1PR3 promotes migration. Certainly, a role of S1PR3 in cell motility has been described for bone marrow-derived mesenchymal stem cells (26), human breast cancer cells (27), and endothelial progenitor cells (28), which are important in tumor angiogenesis. Furthermore, in gastric cancer cells, the balance between S1PR3 and S1PR2 expression is a critical feature to decide whether the cell will increase (S1PR3) or decrease (S1PR2) its migratory capability (29).

Expressions of S1PR4/5 are more limited, and the mechanisms that they regulate are less understood. However, it is known that S1PR4 promotes neutrophil trafficking (30) and dentritic cell differentiation (31), while S1PR5 has been associated with natural killer (NK) cell trafficking (32, 33).

Many studies, in the past few years, have clearly demonstrated that S1P can also function as an intracellular mediator. Dr. Spiegel’s group has shown that S1P binds and modulates the function of different intracellular targets, including tumor necrosis factor (TNF) receptor-associated factor 2 (TRAF2) (34), histone deacetylases, HDAC1 and HDAC2 (35), and prohibitin 2 (36). Recent reports also indicate that S1P binds peroxisome proliferator-activated receptor gamma (PPARγ) in endothelial cells and stimulates angiogenesis (37).

A comprehensive description of S1P signaling is out of the scope of this review, but the reader is encouraged to read some excellent reviews published recently (5, 11, 38).

## The Many Functions of S1P in Cancer

About 20 years ago, two seminal papers described, for the first time, the role of S1P in enhancing proliferation and inhibiting apoptosis (39, 40). Since then, many studies have reinforced a pro-survival function of S1P. On the contrary, ceramide and sphingosine are usually associated with cell growth arrest. The inter-convertibility of these metabolites has led to the concept of “sphingolipid rheostat,” with the balance between S1P vs. sphingosine and ceramide levels, determining cell survival or death (41). Considering its role as growth promoter, it is not surprising that many evidences strongly support a fundamental role of S1P in cancer progression.

### S1P Metabolizing Enzymes in Cancer

It has been demonstrated that S1P levels and SphK1 expression and/or activity are increased in distinct cancer types (42). Table 1 summarizes some of the known cancer types and the biological effects associated to deregulated expression of the enzymes involved in S1P metabolism. Although beyond the scope of this review, these findings have been substantiated by *in vitro* studies using pharmacological and molecular tools. Thus, inhibition of SphK1 activity with corresponding decrease of S1P levels induced apoptosis in acute myeloid leukemia (AML) cells (43, 44), and diminished *in vivo* cell growth of ovarian cancer (45). Importantly, in ovarian cancer, the mTORC1/2 inhibitor WYE-132 reduced SphK1 activity, which induced cytotoxicity and diminished *in vivo* cell growth (45).

**Table 1:** **List of S1P-related proteins deregulated in distinct cancer types**.

Cancer type	Deregulation SphKs/S1PRs	Biological significance	Reference
Acute lymphoblastic leukemia	↑ SphK2 expression	*Mice*: SphK2^−/−^ animals display reduced leukemia. *In vitro*: inhibition of SphK2 suppresses proliferation and induces apoptosis	(46)
Astrocytome	↑ SphK1 expression	*In vivo*: patients exhibit shorter survival time	(47)
Breast cancer ER-negative	↑ SphK1 and S1PR4 expression	*In vivo*: shorter disease-specific survival	(48)
Breast cancer ER-positive	↑ SphK1, S1PR1, and S1PR3 expression	*In vivo*: increase tamoxifen resistance	(49)
Colorectal cancer	↑ SphK1 expression	*In vivo*: increase lymph node and liver metastasis, and advanced TNM stage. *In vitro*: SphK1 knockdown inhibits cell proliferation and invasion	(50, 51)
Gastric cancer	↑ SphK1 expression	*In vivo*: decrease survival	(52)
Glioblastoma multiforme	↑ SphK1, S1PR1, S1PR2, and S1PR3 expression	*In vivo*: poor prognosis and survival in patients. *In vitro*: inhibition of SphK reduces cell viability; inhibition of S1PR1 and S1PR2 diminishes cell migration	(53–55)
Head and neck squamous cell carcinoma	↑ SphK1 expression	*Mice*: SphK1^−/−^ animals display less tumor incidence and volume	(56)
Hepatocellular carcinoma	↑ SphK1/2 and SPL expression; ↓ S1P levels	*In vivo*: poorer tumor differentiation, increase microvascular invasion, and earlier recurrence. *In vitro*: inhibition of SphKs or SPL expression reduces cell proliferation, invasion, and migration	(57)
Large B-cell lymphoma	↓ S1PR2	*Human*: S1PR2 somatic mutations were found in large B-cell lymphoma samples	(58)
Liver cancer	↑ SphK1 expression	*Mice and in vitro*: downregulation of SphK1 inhibits angiogenesis	(59)
Melanoma	↑ SphK1 activity	*Mice*: SphK1 inhibition decreases melanoma cell growth. *In vitro*: SphK1 inhibition retards melanoma cell growth	(60)
Nasopharyngeal carcinoma	↑ SphK1 expression	*In vivo*: SphK1 is related to clinical stage and distant metastasis	(61)
Non-Hodgkin lymphomas	↑ SphK1 expression	*In vivo*: increase clinical grade	(62)
Pancreatic cancer	↑ SphK1 expression	*In vivo*: poor prognosis	(63)
Papillary thyroid cancer	↑ SphK2 expression	*In vivo*: SphK2 expression correlates with clinical stage	(64)
Prostate cancer	↑ SphK1 activity	*Human*: 2-fold increase of SphK1 activity in human prostate Ca section vs. normal counterpart. ↑ PSA, ↑ tumor volume, ↑ treatment failure were associated with increased SphK1 activity	(65)
	Neutralization of S1P	*In vivo*: in orthotopic xenograft model of human PC-3 prostate cancer cells, anti-S1P monoclonal Ab (Sphingomab^®^) inhibited intratumoral hypoxia, induced vascular remodeling and chemotherapy sensitivity	(66)
Thyroid cancer	↑ SphK1 expression	*In vivo*: increased proliferation. *In vitro*: silencing of SphK reduces cell proliferation	(67)
Triple-negative breast cancer	↑SphK1 expression	*In vivo*: decrease patient survival. *In vitro*: knockdown of SphK1 diminishes cell proliferation and migration/invasion	(18, 68)
Uterine cervical cancer	↑SphK1 expression	*In vivo*: aggressive oncogenic behavior, invasion, and lymph node metastasis. *In vitro*: inhibition of SphK1 reduces cell viability and increases apoptosis	(69)
Wilm’s tumor (renal cancer)	↑ S1PR2	*Human*: S1PR2 mRNA overexpressed in Wilm’s tumor samples	(70)

**Cancer type**	**Deregulation of other S1P-related proteins**	**Biological significance**	**Reference**

Colon cancer	↓ SPL and SPP expression	*Human*: SPL expression downregulated in colon cancer tissue. *In mice*: SPL^−/−^ increased susceptibility to CAC. *In vitro*: SPL downregulation diminished stress-induced apoptosis. SPL overexpression had opposite response	(71, 72)
Gastric cancer	↓ SPP expression	*Human*: SPP (protein and mRNA) was downregulated in cancer tissue and correlated with metastasis. *In vitro*: SPP knockdown increased invasion and migration. SPP overexpression induced slow growth and less adhesion	(73)
Glioblastoma	↓ SPP2 and ↑ SphK1 expression	*Human*: increased S1P and decreased ceramide content; high SphK1 and low SPP2 expression in cancer tissue. *In vitro*: Sphk1 inhibition reduced angiogenesis in a coculture model	(74)
Lung cancer	↓ Spns2 expression	*Human*: Spns2 mRNA is reduced in advance lung cancer. *In vitro*: ectopic Spns2 expression induced apoptosis, modulates S1P metabolism and S1PRs expression	(75)
Prostate cancer	↓ SPL expression and activity	*Human*: low SPL and high Sphk1 expression are correlated with aggressiveness and poor prognosis. *In vitro*: SPL downregulation enhanced cell survival after radio and chemotherapy, while SPL overexpression had opposite effects	(76)

Interestingly, microRNAs (miR), which may act as oncogenes or tumor suppressors, also regulate the expression of SphK1. In that regard, miR-506 suppressed tumor angiogenesis through targeting SphK1 mRNA in liver cancer (77), while miR-124 downregulated SphK1 and inhibited proliferation of gastric cancer cells (78). miR-124 expression inversely correlated with metastasis and SphK1 in ovarian cancer, suggesting that downregulation of miR-124 may be a common mechanism to modulate S1P-induced cancer progression (79). Moreover, miR-613 was downregulated in papillary thyroid cancer (PTC) and inversely modulated SphK2 expression *in vitro* and *in vivo* (64). It is likely that regulation of SphKs expression by miR in different tissues will reveal new mechanisms and define improved cancer therapies.

In contrast to the substantial evidences that suggest a crucial role of SphK1 in cancer development, much less is known about the function of SphK2. In that sense, specific inhibition of SphK2 with ABC294640 in colorectal cancer (CRC) cells reduced S1P and increases ceramide levels, thus inhibiting CRC cells and xenografts growth *in vitro* and *in vivo*, respectively (50). Although this action seemed to be receptor-mediated, studies with specific S1PRs agonist or antagonist are lacking. Of great interest, low dose of ABC294640 was able to sensitize cells, making them more susceptible to chemotherapeutic treatment (80, 81). In addition, ABC294640 has been also found to effectively inhibit proliferation and xenograft prostate tumor growth by targeting the androgen receptor and the proto-oncogene myc (81). Indeed, SphK2 was upregulated in acute lymphoblastic leukemia and induced the expression of Myc, suggesting an important role in hematological cancers (46). Downregulation of SphK2 by small interfering RNA also reduced migration of T24 human bladder carcinoma (82), MDA-MB-231 breast cancer, and A-498 kidney carcinoma cells (83). Altogether, these evidences unwrap a new opportunity to consider SphK2 as a potential target, not only to inhibit cancer progression but also to prevent tumor resistance to standard chemotherapy.

Alterations in S1P metabolism and levels are not only regulated by SphKs but also by SPPs and SPL. In line with the cancer-supporting role of S1P, reduced expression of SPPs led to augmented S1P levels and is also a common feature of different tumors. In fact, downregulation of SPP1 in gastric cancer tissues enhanced metastasis (73), suggesting that SPP1 expression may serve as a prognostic marker in gastric cancer that correlates with patient’s survival (73). Also, the increased S1P content detected in human glioblastoma tissue was associated with SphK1 expression but inversely correlated with SPP2 expression, suggesting that the shift of the S1P rheostat may play a role in the development of this tumor (74). SPL is downregulated in prostate and colon cancer (71, 72, 76), and it was shown to have an implication in chemo and radiotherapy resistance (76), implying that regulation of SPL activity might be a novel approach to cancer treatment. Furthermore, deletion of SPL in intestinal epithelium provoked an increased incidence of colitis-associated cancer and enhanced inflammatory response in mice (72). Interestingly, the expression of the specific S1P transporter, Spns2, was also reduced in advance lung cancer patients (75). On the contrary, over expression of Spns2 induced apoptosis in non-small cell lung cancer (NSCLC) cells (75). Although it is clear that the evidences are less abundant in comparison with SphKs, these findings suggest that the modulation of S1P-degrading enzymes and S1P transporter might constitute a new therapeutic option.

Even an overwhelming amount of data indicate that increased S1P levels were associated with cancer progression, many recent reports showed disconcerting results: (i) in patients with hepatocellular carcinoma, a decrease in S1P levels in HCC tissue was linked to less tumor differentiation and more microvascular invasion (57); (ii) specific inhibition of SphK1 in 1483 head and neck carcinoma cells with PF-543 did not affect cell proliferation or survival (84), and (iii) pharmacological and molecular inhibition of SphK1 and SphK2 did not affect tumor cell growth, both *in vitro* and *in vivo* (85). The discrepancies of these results with many opposing evidences warrant further investigation. Nonetheless, it is important to highlight that PF-543 exerted anti-proliferative effects in diverse CRC cell lines, suggesting differential and tissue-dependent functions for SphK1 (86).

### S1PRs and Cancer

Similar to SphKs, deregulation of S1PRs expression is frequently observed in many cancer types and may account for significant differences in tumor angiogenesis and invasiveness. In glioblastoma multiforme (GBM), one of the brain tumors with worse prognosis, increased expression of S1PR1 and S1PR2 correlated with decreased patient’s survival (53). In human pancreatic cells, S1P also increased proliferation and migration through a mechanism that involves activation of c-Src pathway (87). A very recent report showed that activation of S1PR2 in epithelial cells was crucial for elimination of neighboring cancer cells, a process known as epithelial defense against cancer (EDAC) (88). Of great interest, the process involved a gradual accumulation of Filamin A (FlnA), an actin-binding protein that we recently demonstrated to modulate S1P-induced NF-κB activation in melanoma cells (89).

A central role of S1P in tumor progression has been further highlighted by the development of Sphingomab^®^, a neutralizing anti-S1P monoclonal antibody (90) that prevents signaling through all S1PRs. Exciting studies in xenograft models showed that Sphingomab reduced blood vessel formation and tumor-associated angiogenesis (90). Furthermore, this antibody blocked hypoxia-inducible factor 1α (HIF-1α) accumulation in low-oxygen environments and modified vessel architecture, leading to an improved sensitivity to chemotherapeutic drugs in an *in vivo* model of prostate cancer (66) and sunitinib-resistant renal cancers (91).

More recently, the development of Spiegelmers^®^, synthetic oligonucleotides built of non-natural L-nucleotides, has opened new opportunities to target S1P. Indeed, NOX-S93 is a recently identified Spiegelmer^®^ that binds S1P in the low nanomolar range and blocks the angiogenic activity of the lipid and vascular endothelial growth factor (VEGF) (92). Moreover, preclinical data indicate that NOX-S93 reduces S1P-induced metastasis of Rhabdomyosarcoma (RMS) (93). Thus, strong evidences suggest that S1P inhibition may be a prospective strategy to deprive cancer cells from basic nutrients and diminish tumor growth and chemotherapy resistance.

### S1P Is a Putative Prognostic Factor

Sphingosine-1-phosphate has recently gained additional significance through many reports, suggesting that it may serve as a prognostic factor in different kind of tumors. In fact, increased SphK1 expression was associated with tumor size, lower survival, recurrence and poor prognosis in HCC, astrocytoma, and breast cancer patients (47, 48, 94). Moreover, in uterine cervical cancer, SphK1 expression was correlated with invasion and lymph node metastasis (69). Intriguingly, plasma levels of S1P were lower in prostate cancer patients than in healthy controls, perhaps due to the reduced expression of SphK1 subsequent to decreased hematocrit featured in cancer patients (95). There is no doubt that substantiation of these findings in other types of cancers may shed some light about possible clinical implications and use of S1P as a cancer biomarker.

### S1P and Cancer Chemoresistance

Accumulating evidences imply that S1P may have a potential role in cancer chemoresistance, one of the main causes of poor treatment outcome and tumor relapse. For instance, CRC cells with acquired resistance to cetuximab, an epidermal growth factor receptor (EGFR) inhibitor, overexpressed SphK1, and its inhibition re-established sensitivity to the drug. These findings were corroborated in CRC patients whose SphK1 overexpression also resulted in resistance to cetuximab (96). Similarly, both expression and activity of SphK1 were increased in sunitinib-resistant renal cell lines (97), and a gene-expression analysis of different tumor cell lines resistant to oxaliplatin, cisplatin, and docetaxel identified that drug resistance was related to SphK1 expression (98). These evidences support the relevance of ongoing clinical trials with combinational therapies of safingol (SphK1 inhibitor) and classic chemotherapeutic drugs.

In agreement, many reports suggest that other proteins involved in S1P signaling may also serve as potential targets to overcome resistance to chemotherapy. Thus, SphK2 overexpression has been correlated to gefitinib chemoresistance in NSCLC cells (99), and to proliferation of chemoresistant hormone-independent breast cancer (80), while in cisplatin-resistant melanoma cells, combined treatment with the S1PR modulator FTY720 and cisplatin induced cell death (100).

Indeed, increase in S1P levels seems to be crucial to chemotherapy resistance. Thus, preventing S1P degradation by SPL silencing in prostate cell lines enhanced survival after chemotherapy and radiation (76). In agreement, levels of S1P were also increased in metastasic sites of RMS cells inoculated in mice treated with chemotherapy or γ-irradiation (93). Therefore, S1P may be important in establishing a microenvironment that facilitates metastasis after standard tumor treatments.

Taken together, these data strongly support the “sphingolipid rheostat” concept and indicate that modulation of S1P, ceramide, and sphingosine levels may constitute a promising anti-cancer therapy, but it also suggests that controversy still exists pertaining to the exact role of S1P in tumor progression. In that sense, it is possible that differential functions of S1P in distinct cancer types may be related not only to the regulation of the tumor cell but also to its role as a factor present in the tumor microenvironment.

## S1P as a Modulator of the Tumor Microenvironment

Since Virchow proposed that tumors behave like wounds that do not heal (101), a link between inflammation and cancer has been extensively studied, but it was not widely understood until recently (102). The tumor microenvironment is populated by tumor cells and non-neoplastic stromal cells as lymphocytes, macrophages, dendritic cells (DC), fibroblasts, mast cells (MC), and endothelial cells, among others. They can interact by direct cell–cell contact or they may communicate through soluble factors. Indeed, many evidences demonstrate that the tumor microenvironment has an important role in allowing the tumor to express its full neoplastic phenotype by increasing angiogenesis and metastasis.

### Monocyte Recruitment and Macrophage Polarization

Inflammatory infiltrates are often a hallmark of many cancers, leading to sustained release of proinflammatory, anti-inflammatory, and immunosuppressive cytokines in the tumor microenvironment. While the initial purpose of these inflammatory cells is to fight the tumor, at the end, they may enhance cancer progression. The recruitment of macrophages to the tumor is dependent on chemokines secreted by the tumor and represents a delicate balance between the antitumor response and the production of mediators that may facilitate the growth of the tumor. Accordingly, a broad range of macrophage subsets has been described. M1 (classically) or M2 (alternatively activated) macrophages constitute the extremes of the scale. M1 macrophages kill tumor cells, whereas M2 macrophages produce angiogenic factors, anti-inflammatory cytokines, and stimulate tumor growth (103). Tumor-associated macrophages (TAM) display an M2-like phenotype and a correlation between TAM density and poor prognosis of many cancers has been clearly established. Moreover, new evidences connect TAMs with chemotherapy resistance.

**Figure 2:**
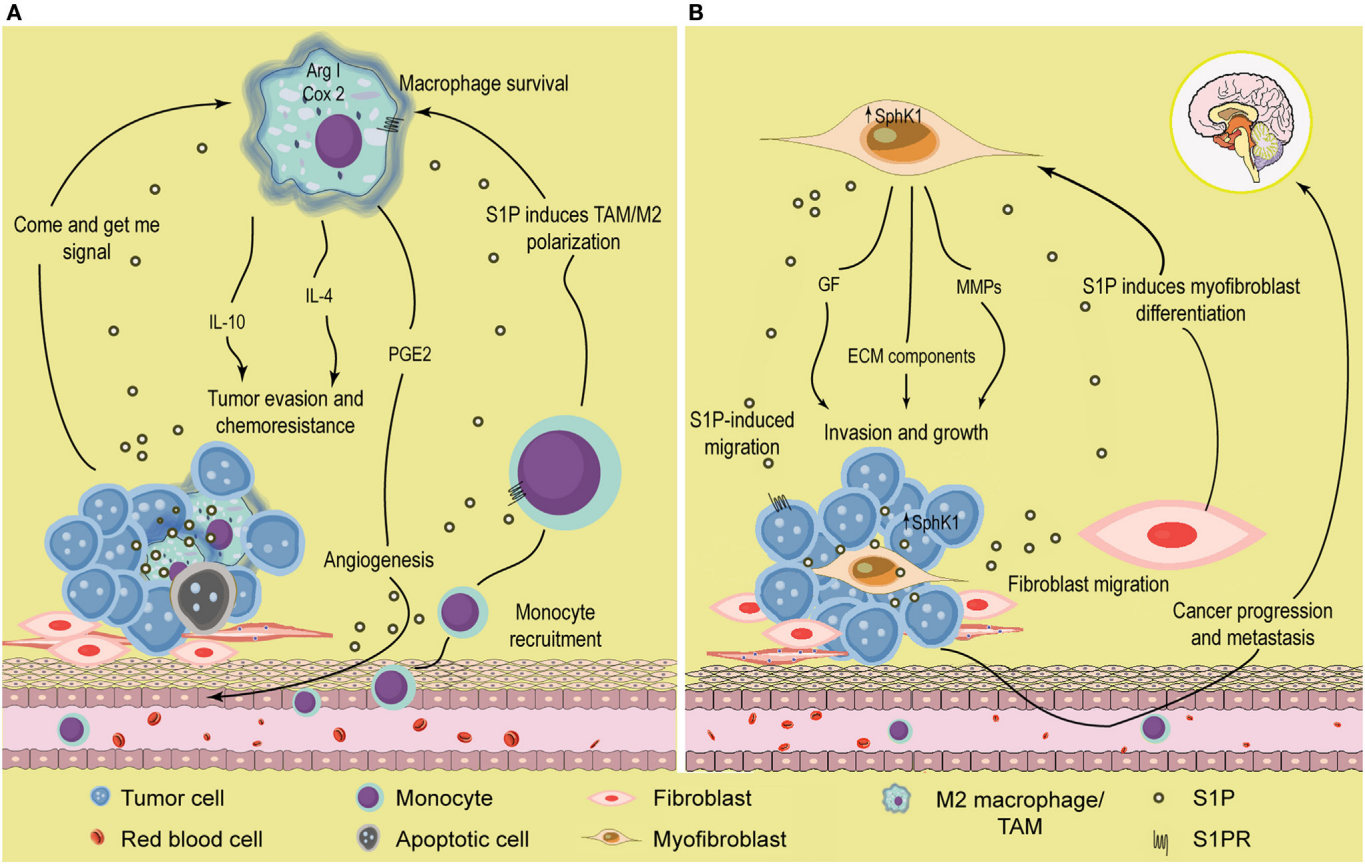
**Role of S1P in the tumor microenvironment**. **(A)** S1P induces monocyte recruitment and macrophage polarization. Apoptotic or tumor cells release S1P that binds S1PR1 and recruits circulating monocytes toward the tumor microenvironment. Once they enter the tissues, monocytes differentiate into macrophages with distinct phenotype according to the surrounding signals. In the tumor microenvironment, S1P exhibits many functions; (i) increase macrophage survival; (ii) function as a “come and get me signaling” of dying cells to attract and enhance macrophage migration through the binding of S1PR1, and (iii) stimulate TAM/M2 macrophage polarization by activating S1PR1/2/4. TAMs are characterized by increased Arginase I (Arg I) activity and secrete the anti-inflammatory cytokines IL-4 and IL-10 that further contribute to induce a permissive microenvironment characterized by tumor evasion and chemotherapy resistance. In addition, S1P released from apoptotic cells activates S1PR1/3 in macrophages to upregulate the expression of cycloxoygenase 2 (Cox-2) and stimulate the secretion of prostaglandin E2 (PGE2) that support migration of endothelial cells and angiogenesis, a hallmark of tumor progression. **(B)** S1P modulates the interaction between tumor and stromal cells. SphK1 expression is upregulated in melanoma cells, increasing the production of S1P. Melanoma cells stimulate the recruitment of dermal fibroblast toward the tumor microenvironment, and S1P induces the differentiation to myofibroblast/cancer-associated fibroblast (CAF). In turn, CAFs display increased SphK1 expression and release S1P that enhances melanoma migration (through S1PR3) and growth. CAF also express α-smooth muscle actin (α-SMA) and secrete growth factors, extracellular matrix (ECM) components, and metalloproteinases (MMP) that augment cancer progression and promote tumor metastasis.

Sphingosine-1-phosphate has been largely recognized as a chemoattractant lipid for many cell types, including inflammatory cells (104, 105). Indeed, we have shown that S1P released from apoptotic cancer cells, acted as a “come and get me” signal and attracted monocytes to almost the same extent as monocyte chemoattractant protein-1 (MCP-1/CCL2) (106) (Figure 2A). Once they reach the tissue, monocytes differentiate into macrophages. Interestingly, apoptotic cells also regulate macrophages by enhancing expression of S1PR1, which in turn is required to increase motility (107) (Figure 2A). It is well known that apoptotic cell clearance is an important regulatory mechanism that maintains homeostasis; thus, deregulation of this activity results in chronic inflammation characteristic of cancer among other diseases. The role of S1P as a signal from apoptotic cells has been well documented in the last years in many models, including erythropoiesis (108), breast cancer (109, 110), kidney repair (111), and acute T cell leukemia (107, 112, 113). Mechanistically, while some reports indicated that increased release of S1P from apoptotic cells was related to SphK1 activation (106), others suggested that S1P was mainly derived from SphK2 (113). These opposite results may be due to the use of different agents to induce apoptosis: doxorubicin and SphK inhibitors induced a dramatic increase in SphK1 expression (106), while staurosporin treatment supported SphK2-induced release of S1P (113).

Notably, release of S1P from apoptotic cells has dramatic implications in macrophage polarization, cytokine release, and angiogenesis. Thus, S1P polarized macrophages toward an M2 phenotype (110), increased release of IL-10, a distinctive anti-inflammatory cytokine, from TAMs (109, 110), and stimulated macrophages to secrete prostaglandin E2 (PGE2) that, in turn, induced migration of endothelial cells increasing angiogenesis, a seal of tumor progression (112) (Figure 2A). S1P released from apoptotic cells also induced Bcl-X(L) and Bcl-2 upregulation and protected macrophages from cell death (113). In that regard, it is attractive to speculate that S1P has a dual role on macrophages: (i) cell death inhibition and (ii) induction of M2 polarization, both supporting cancer progression. Importantly, *in vivo* xenograft experiments suggested that growth and M2 macrophage polarization, but not total infiltration, were compromised in mice injected with MCF-7 breast cancer cells carrying a short hairpin (sh)RNA plasmid to downregulate SphK2, strongly suggesting a role for this isoform in S1P-mediated macrophage growth and M2 polarization (114). However, it has also been suggested that increased expression of SphK1 may serve as an M2 marker (115).

Although the evidences indicated above clearly support a direct role of S1P as a modulator of TAMs, recent finding suggests that its function may be even more complex than expected and may regulate the function of different ligands. In that sense, S1PR1 signaling was also required for Angiotensin II (Ang II)-dependent production of TAMs in the spleen (116). To further emphasize its role as modulator of macrophage functions, direct S1P stimulation also induced M2 polarization through IL-4 secretion and signaling (117) and S1PR1-mediated decrease of proinflammatory cytokines secretion with simultaneous inhibition of inducible nitric oxide synthase (iNOS) activity and augmented arginase I activity (118) (Figure 2A).

The assumption that S1P is important for both monocyte recruitment and switch toward a less aggressive macrophage phenotype may be of great utility for cancer cells to generate a permissive microenvironment and, at the same time, to evade the tumor-killing response elicited by cytotoxic macrophages. In this regard, S1P may not only promote cancer cell growth but also decrease the immune response that destroys the tumor. Importantly, S1P-induced attenuation of NF-κB activity in TAMs (118), but not in cancer cells, may be another mechanism of the tumor to elude the immune response of the host. On the other hand, although S1P-mediated induction of M2 macrophage polarization and angiogenesis correlates with cancer progression, it may also prove to be useful for bone regeneration and physiological situations that require wound repair (119). Thus, it is clear that targeting S1P as a macrophage modulator involves establishing a compromise between beneficial and harmful outcomes. The identification of the potential functions of S1P as master regulator of macrophage production and polarization is perhaps one of the most challenging tasks for this field in the coming years. Indeed, reprogramming TAMs phenotype to activate antitumor response is a proposed strategy for cancer treatment that warrants further clinical evaluation.

### Resident Mast Cell Functions and Phenotypic Plasticity

Mast cells are tissue-dwelling cells, thus, *de facto* components of the tumor microenvironment and endowed with immuno-modulating functions through the production of numerous mediators, including S1P (120, 121). Human and mouse studies have established pro- or antitumorigenic functions for MC (122). Increased number of MC has been observed in all solid tumors, such as melanoma (123), prostate (124–127), colorectal (128) and pancreatic (129) adenocarcinomas, and NSCLC (130). Confusingly, MC elevation has been described as indicative of a poor (124, 127) or a good (125, 126) prognosis. For example, in prostate cancer, increased number of MC around, but not within, cancer foci positively correlated with advanced stage (124), whereas high intratumoral MC density was accompanied with a good prognosis (126). Thus, it is suggested that MC functions are likely to be determined by the organ affected with and the stage of tumor, in addition to their location in the tissues relative to tumor foci (122).

Mast cells may differentiate into two subsets depending upon the tissue they populate and the microenvironmental conditions, as defined by the protease repertoire harbored within cytoplasmic granules: connective tissue or serosal MC express tryptase and chymase, whereas mucosal MC are single positive for tryptase (120). Importantly, MC-derived proteases can activate matrix metalloproteinases (MMP) that are critical to tumor growth and metastasis, and we recently reported that S1P-mediated MC activation triggered substantial release of MMP and pro-angiogenic VEGF (131). We and others have shown that MC can produce S1P (3, 132). Therefore, it is tempting to speculate that MC could be one of the initial and prime cellular sources of local S1P in tissues that may trigger tumorigenesis.

It is noteworthy that augmented levels of S1P may act in a paracrine manner on the neighboring cells and also in an autocrine manner on MC, leading to further MC activation. Our laboratory has discovered key contributions of MC to the inception and perpetuation of multi-organ manifestations of inflammation, including tissue remodeling, perivascular edema, and chemoattraction of T lymphocytes through signaling of S1P (105, 120, 133). As previously mentioned, a link between chronic inflammation and cancer was originally described more than 100 years ago, and MC could orchestrate the transition from inflammation to cancer. Interestingly, we discovered that exposure to S1P led to human MC hyperplasia and chymase expression, in addition to MC activation (121), and chymase-expressing MC were hyperactive with increased mediator content and release and extended surface receptor expression (121, 134), also reported by others (135).

In sum, the presence of MC in tissues prior to cancer inception and their ability to produce and respond to S1P by secretion of tumor-influencing mediators may qualify MC as key regulators of the tumor microenvironment, pointing toward the MC/S1P axis as a promising interventional target to prevent cancer initiation and progression.

### S1P Functions in Other Immune Cells

Even though most of the literature point to a crucial role of S1P in monocytes/macrophages recruitment, survival, and polarization, it has also been suggested that S1P modulates the function of other immune cells of the tumor microenvironment. Thus, while S1P acts as a chemoattractant of NK cells, it also inhibits its cytotoxic activity through a mechanism that involves the increase of cAMP levels and activation of protein kinase A (PKA) (136). Moreover, although Rolin et al. reported that S1P does not affect the cytotoxic activity of NK cells, they showed that S1P protects human myeloid leukemia K562 cells from NK cells-induced lysis through the activation of S1PR1 (137). Whereas these evidences indicate that S1P may contribute to tumor evasion from NK cells, on the other hand, S1P also inhibits NK-mediated cell lysis of immature monocyte-derived DCs (137), which may favor antigen presentation to T cells. Moreover, S1P enhances endocytosis and migration of mature DCs through S1PR3 engagement (138), an event that may increase immune response toward cancer cells. In addition, in the presence of S1P, monocytes differentiate into DCs that do not express CD1a and display reduced capability of stimulating T lymphocytes as compared with DCs that matured in the absence of S1P (139). Consequently, this duality in the role of S1P in NK and DCs in migration and phenotypic modulation deserves more attention to unequivocally establish its patho-physiological relevance.

The role of S1P on lymphocyte migration and egress from lymphoid organs has been extensively studied and resulted in the development of the S1PR1 functional agonist fingolimod for the treatment of autoimmune diseases such as MS (140). However, in the last years, many reports suggest distinct and, in some cases, conflicting functions of S1P in B and T lymphocytes regarding cancer progression. Differential expression of S1PRs has a crucial function in B and T cell lymphocyte migration and activation. In diffuse large B-cell lymphoma (DLBCL) cell lines, expression of S1PR2 reduces tumor growth and is a good prognosis factor for patient survival (141). In addition, the modulation of S1PR1, S1PR2, and S1PR4 engagement regulates B cell circulation in patients with chronic lymphocytic leukemia (CLL) (142). Thus, while S1PR1 is expressed at low levels in CLL lymph nodes as compared with normal B cells (143), increased expression of S1PR1 correlates with signal transducer and activator of transcription 3 (STAT3) activation and survival in B-cell lymphoma (144). Furthermore, decreased expression of S1PR1 in CLL B cells impairs their egress from the peripheral lymphoid organs and enhances their survival (145).

Regulatory T cells (Tregs) have a crucial function in cancer progression since they suppress the antitumor activity of other immune cells. While fingolimod inhibition of Tregs proliferation may abrogate the suppressive role of these cells in the tumor microenvironment (146), it has more recently been shown that S1PR1 signaling activates STAT3, resulting in accumulation of Tregs and tumor growth in an orthotopic model of breast cancer (147). In that regard, the elucidation of the subjacent mechanisms involved in these opposite functions of S1P may be of great importance to overcome the immunological tolerance frequently observed in cancer progression.

In sum, in the last few years, it has become clearly evident that S1P modulates numerous aspects of immune cells. However, the discrepancy and complexity of its actions guarantee that more studies are needed to establish the role of this lipid in the immune cells that inhabit the tumor microenvironment.

### S1P and Cancer-Related Inflammatory Pathways

A role of S1P in regulating NF-κB and STAT3 activation, two key signaling pathways that link cancer with inflammation, has been long suspected, but only recently uncovered. It has been reported that S1P can activate NF-κB through both intracellular and extracellular mechanisms. Receptor-mediated activation involved S1PR1/3 in different cell types (115, 148–153). Moreover, we have recently demonstrated that activation of NF-κB by extracellular S1P in melanoma cells involved both S1PR1 and S1PR2 and was inversely correlated with the expression of the actin-binding protein FlnA (89). Interestingly, FlnA physically interacts with SphK1 (16) and TNF receptor-associated factor 2 (TRAF2) (154). Certainly, we established that intracellular S1P generated by SphK1 was a required cofactor for TRAF2 E3 ubiquitin ligase activity, linking TNF signaling to NF-κB activation in melanoma cells (34). Our results were supported by other reports showing that TRAF-interacting protein (TRIP), a cellular-binding partner of TRAF2, abrogated TNF-induced NF-κB activation by inhibiting binding of S1P to TRAF2 and thus suppressing its E3 ubiquitin ligase activity (155). Altogether, it is clear that S1P is able to modulate, in distinct ways, the activation of NF-κB providing a link between chronic inflammation and cancer (156). Indeed, NF-κB activation in macrophages also promoted the switch toward an M2 phenotype (157). Considering the role of S1P in M2 macrophage polarization, it will be interesting to determine whether or not the NF-κB-induced anti-inflammatory switch in macrophages could be triggered by S1P present in the tumor microenvironment.

A connection between S1P and STAT3 activation has also been shown to be critical for tumor progression. A pivotal study established a direct relationship between S1PR1 and STAT3 expression in distinct tumors, including lymphoma, adenocarcinoma, melanoma, breast, and prostate cancer (158). These findings were further reinforced later to show that silencing of S1PR1 expression diminished expression of STAT3-regulated genes and inhibited tumor progression (144). Moreover, Silva et al. showed that in a mice model of cancer-induce anorexia, high levels of S1PR1 were correlated with augmented phosphorylation of STAT3 in the hypothalamus (159). Interestingly, treatment with FTY720 does not affect tumor growth, but reduces weight loss and increases survival. The link between S1P and STAT3 is not restricted to S1PR1; thus, pharmacological inhibition of SphK2 abrogated STAT3 phosphorylation, leading to decreased proliferation of cholangiocarcinoma cells (160), while in ER-negative breast cancer cells, SphK1 knockdown led to a significant reduction in leptin-induced STAT3 phosphorylation (161).

Over the last years, accumulating evidences have demonstrated that S1P signaling was crucial for persistent activation of STAT3 in epithelial/tumor cells in inflammation-associated colon cancer (162). Thus, targeted deletion of SPL in normal intestinal epithelial cells, which increases S1P levels, enhanced colitis-associated cancer through STAT3-modulated regulation of proinflammatory cytokines (72). Moreover, silencing of SPL in fibroblast also supported tumor progression. In an elegant study, Liang et al. demonstrated that S1P, derived from increased SphK1 expression in CRC, drove a malicious loop that involved NF-κB activation and IL-6 production with the subsequent induction of STAT3 and upregulation of S1PR1 (163). This mechanism is crucial to connect chronic inflammation with colon cancer.

### S1P Regulates Interactions between Different Cells from the Tumor Microenvironment

The role of S1P in the tumor microenvironment has been recently highlighted by different studies that described how this lipid may modulate interactions between distinct cell types in the tumor. Thus, expression of SphK1 in dermal fibroblasts enhanced tumor growth in a model of melanoma (164) (Figure 2B). In addition, SphK1-expressing melanoma cells secreted factors required for fibroblasts to myofibroblasts differentiation, strongly indicating that SphK1 was crucial for communication between stromal and cancer cells in melanoma (Figure 2B). Reciprocally, myofibroblasts released S1P and metalloproteinases that increased melanoma growth and metastasis, respectively (Figure 2B). S1P also mediates mutual interactions in the pancreas between tumor and stromals cells, leading to tumor progression (165). Indeed, pancreatic cancer cells overexpress SphK1 and secrete S1P which, in turn, binds to S1PR2 and induces stromal cells to release MMP-9, in a mechanism controlled by NF-κB activation. This feed-forward loop further enhanced tumor cell migration and invasion *in vitro* and cancer growth *in vivo*. Also, Beach et al. (166) established that SphK1 acted as a critical mediator of differentiation and of TGF-β-induced activation of cancer-associated fibroblasts, a cell type that inhabits the tumor microenvironment and supports cancer progression. It was recently demonstrated that communication between cancer and stromal cells was dependent on systemic host-derived S1P rather than S1P generated in tumor cells (167). Importantly, only systemic S1P regulates lung metastasis. Altogether, these evidences strongly support that S1P may facilitate the communication between malignant and stromal cells to enhance tumor development.

### Role of S1P in Hypoxia

The establishment of the appropriate microenvironment is decisive for survival of cancer cells. Hereof, hypoxia, a condition where the tissues are not adequately oxygenated, is a typical feature of solid tumor microenvironment (168, 169). Hypoxia is a consequence of increased oxygen consumption by abnormally proliferating cancer cells that triggers the formation of new atypical blood vessels resulting in defective blood perfusion. Interestingly, hypoxia may stimulate or inhibit proliferation depending on the cell type.

**Figure 3:**
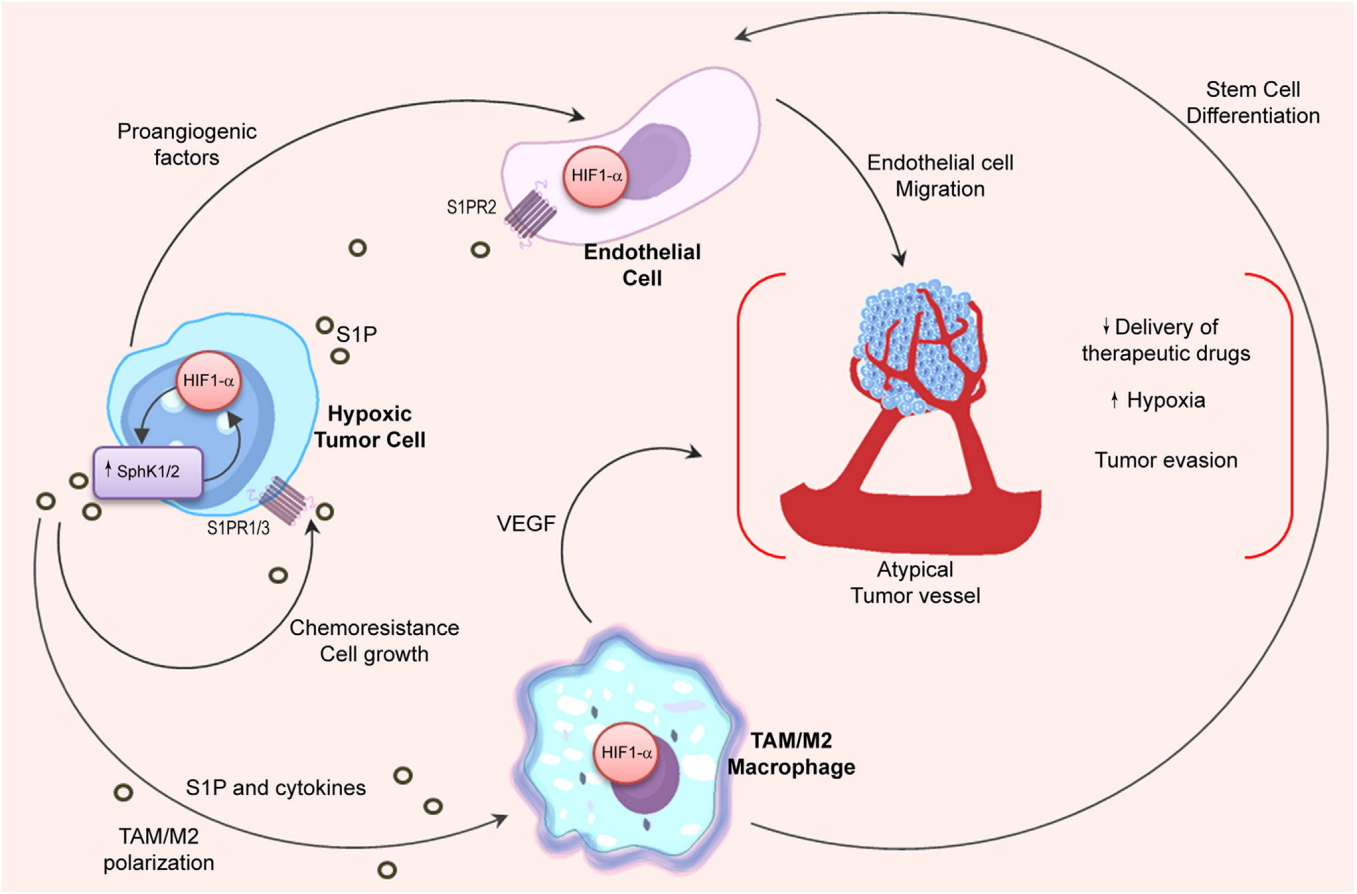
**Relation between S1P and hypoxia in the tumor microenvironment**. Angiogenic switch is crucial for tumor progression. HIF1α stability in hypoxic tumor cells is associated to SphK1 expression, which is also regulated by HIF1α. In the tumor microenvironment, hypoxic cells release S1P and cytokines (oncostatin M, eotaxin, and IL-6) to induce M2 macrophage/TAM polarization. In turn, stabilization of HIF1α in TAMs is important to stimulate stem cell differentiation and to release VEGF that supports angiogenesis. Importantly, S1P production in hypoxia increases endothelial cell migration through S1PR2 engagement and enhances the development of new vessels with deficient architecture that impairs the delivery of chemotherapeutic drugs.

The oxygen-sensitive transcription factor hypoxia-inducible factor 1 alpha (HIF1α) is the master regulator of the hypoxic response, and its expression is mainly regulated at the posttranslational level (168). Interestingly, many evidences indicate that S1P can regulate the activity and expression of HIF1α (170) (Figure 3). In that regard, it has been shown that SphK1 and S1PR2 were required to stabilize HIF1α in different cell types (171, 172). Although most of the literature indicates that S1P is involved in HIF1α regulation, it has also been described that SphK1 activity may control HIF2α expression and transcriptional activity through a phospholipase D (PLD)-driven mechanism in clear cell renal cell carcinoma (173).

Notably, hypoxia also sustains M2 macrophage polarization (174, 175). Considering that apoptotic cells release S1P (106, 110) that has been associated to HIF1α activation in macrophages (176), it is tempting to hypothesize that macrophage’s switch in hypoxia may be regulated by S1P signaling. Indeed, inhibition of S1P not only reduced hypoxia *in vivo* but also modified the structure of intratumoral vessels resulting in enhanced delivery of chemotherapeutic drugs (66). To emphasize the interconnection between the S1P and hypoxia pathways, it has been shown that S1P enhanced endothelial CD31-positive cell differentiation (176), while hypoxia induced S1PR1 expression thus increasing migration of endothelial cells and neovascularization (177).

Recent findings suggest that the role of S1P in hypoxia can be more extensive than previously thought and may involve multiple pathways and mechanisms. In that regard, in glioma cells, while silencing of HIF2α reduced expression of SphK1 and S1P levels, downregulation of HIF1α increased SphK1 (178). Moreover, SphK2 activity was increased in A549 lung cancer cells cultured in hypoxia resulting in secretion of S1P that, in turn, protected against apoptosis and induced chemoresistance (179).

Although still warranting further studies, these results suggest that targeting S1P signaling in hypoxic conditions may be a potential mechanism to decrease angiogenesis and overcome resistance to chemotherapy.

## S1P and Cancer Therapeutics

While many evidences in cellular and animal models suggest that targeting S1P axis may be of clinical benefit in cancer treatment, some compounds have only recently been utilized in clinical trials.

Likely, the most promising therapy involves Sonepcizumab (Asonep), the humanized version of the sphingomab antibody that specifically targets S1P. Sonepcizumab has recently completed Phase I clinical trials for treatment of solid tumors (NCT00661414).

Importantly, although many SphK1 inhibitors were shown to decrease angiogenesis, tumor growth, and proliferation (180), only one Phase I clinical trial with Safingol (SphK1 inhibitor) in combination with Cisplatin has been completed (NCT00084812) and indicated absence of toxicity (181). It is important to note that Safingol not only inhibits Sphk1 but also PKC, although with a slightly higher K_i_.

Even though PF543, the most potent and selective SphK1 inhibitor described to date, decreased S1P levels, it had no effect on cancer cell viability (180), which discouraged the initiation of clinical trials with this compound. However, recent findings showing that PF-543 suppressed CRC xenograft growth and improved mice survival (86) may support renewed translation efforts. Of note, a Phase I clinical trial has already been completed with ABC294640 (SphK2 inhibitor) in patients with solid tumors (NCT01488513) and refractory/relapsed DLBCL (NCT02229981), but no results have been reported to date.

Considering the distinct functions of S1P in normal physiology, it is apparent that further studies will be needed to establish the safety and adverse effects associated with targeting the S1P axis.

## Concluding Remarks

In the last few years, it has become clear that S1P exerts dual functions and may modulate both cancer and stromal cells. The role of S1P in cancer is not limited to enhance tumor growth, viability, and metastasis but S1P may also modulate the functional phenotype of immune cells that surround the tumor, which, in turn, may initiate bidirectional communication in the tumor microenvironment orchestrating cancer progression and chemoresistance. Thus, it is imperative to consider the tumor microenvironment as a key player when designing new potential therapies to overcome pitfalls associated with the current treatments of many human cancers.

## Author Contributions

YR reviewed the literature, wrote the first draft, organized the figures/tables, and revised critically the article. LC, MC, and AA reviewed the literature, drafted some parts, and revised critically the manuscript. CO and SA reviewed the literature, prepared the final version, made substantial contributions to the conception of the review, and revised critically the article. All the authors read and approved the final version of the manuscript.

## Conflict of Interest Statement

The authors declare that the research was conducted in the absence of any commercial or financial relationships that could be construed as a potential conflict of interest.
